# Spontaneous or Traumatic Intratumoral Hemorrhage? A Rare Presentation of Parafalcine Meningioma

**DOI:** 10.7759/cureus.11486

**Published:** 2020-11-14

**Authors:** Danielle D Dang, Luke Mugge, Omar Awan, John Dang, Mahesh Shenai

**Affiliations:** 1 Neurological Surgery, Inova Hospital System, Falls Church, USA; 2 Internal Medicine, Walter Reed National Military Medical Center, Bethesda, USA; 3 Neurological Surgery, Inova Health System, Falls Church, USA

**Keywords:** meningioma, traumatic brain injury, intratumoral hemorrhage, intracranial hemorrhage

## Abstract

While hemorrhage is commonly encountered in various intracranial tumors, it is relatively rare in benign meningiomas. We present an interesting case report of a 75-year-old male who fell during intoxication, sustaining right frontal cranial trauma, coincidentally directly overlying a previously undiagnosed right frontal meningioma. He experienced an acute neurological decline and was found to have an intracranial hematoma, causing significant mass effect and herniation. Based on the radiographic appearance, an underlying neoplasm with significant edema was suspected. Intraoperatively, the hematoma and mass were successfully evacuated, and post-operative pathology confirmed the presence of a World Health Organization Grade I meningioma with a microcystic and angiomatous pattern. We speculate on the mechanism of injury and hemorrhage in this patient through review of the literature and discussion of medical and pathological risk factors.

## Introduction

Meningiomas are neoplasms theorized to arise from the arachnoid cap cells, which surround and support the brain and spinal cord. Though comprising almost one-fifth of all primary intracranial tumors, intratumoral hemorrhage associated with meningioma is markedly rare [1]. For comparison, the rate of tumor-related intracerebral hemorrhage is estimated at up to 4.4% of all presentations of intracranial hemorrhage, yet spontaneous hemorrhage due to underlying meningioma only occurs in 0.5 to 2.4% of these benign lesions [2,3]. In fact, in the two largest series analyzing the natural history of meningiomas, no presentation or development of associated hemorrhage was noted [4]. When occurring, hemorrhage can manifest as a subarachnoid hemorrhage, subdural hematoma, and/or intratumoral hemorrhage and is subsequently associated with an increased mortality rate, estimated to be between 28-55% [2,4,5]. Given the known vascularity of such lesions, several contributing mechanisms for hemorrhage have been suggested but remain poorly understood. Etiologies proposed include compensatory hypertrophy of feeding vessels, rapid and abnormal angiogenesis producing friable vessel walls, systemic processes increasing vascular fragility including hypertension and diabetes mellitus, direct vascular invasion by tumor cells, extensive intratumoral necrosis and infarction, stretching and rupture of subdural veins, and minor trauma [1,6,7]. Furthermore, recent studies have proposed potential risk factors for hemorrhagic presentation, including: age >70 or <30 years, intraventricular or convexity location, traumatic brain injury, concomitant anticoagulation, serotonin-modulating therapy, and/or high-dose estrogen replacement, and malignant-appearing, translational, or angiomatous histopathology [1,8].

In this report, we describe a unique case of an intratumoral hemorrhage within a frontal parafalcine meningioma, arising from a fall producing a direct frontal impact. To our knowledge, this is the first report of an intratumoral hemorrhage caused by direct impact trauma.

## Case presentation

Clinical presentation

**Figure 1:**
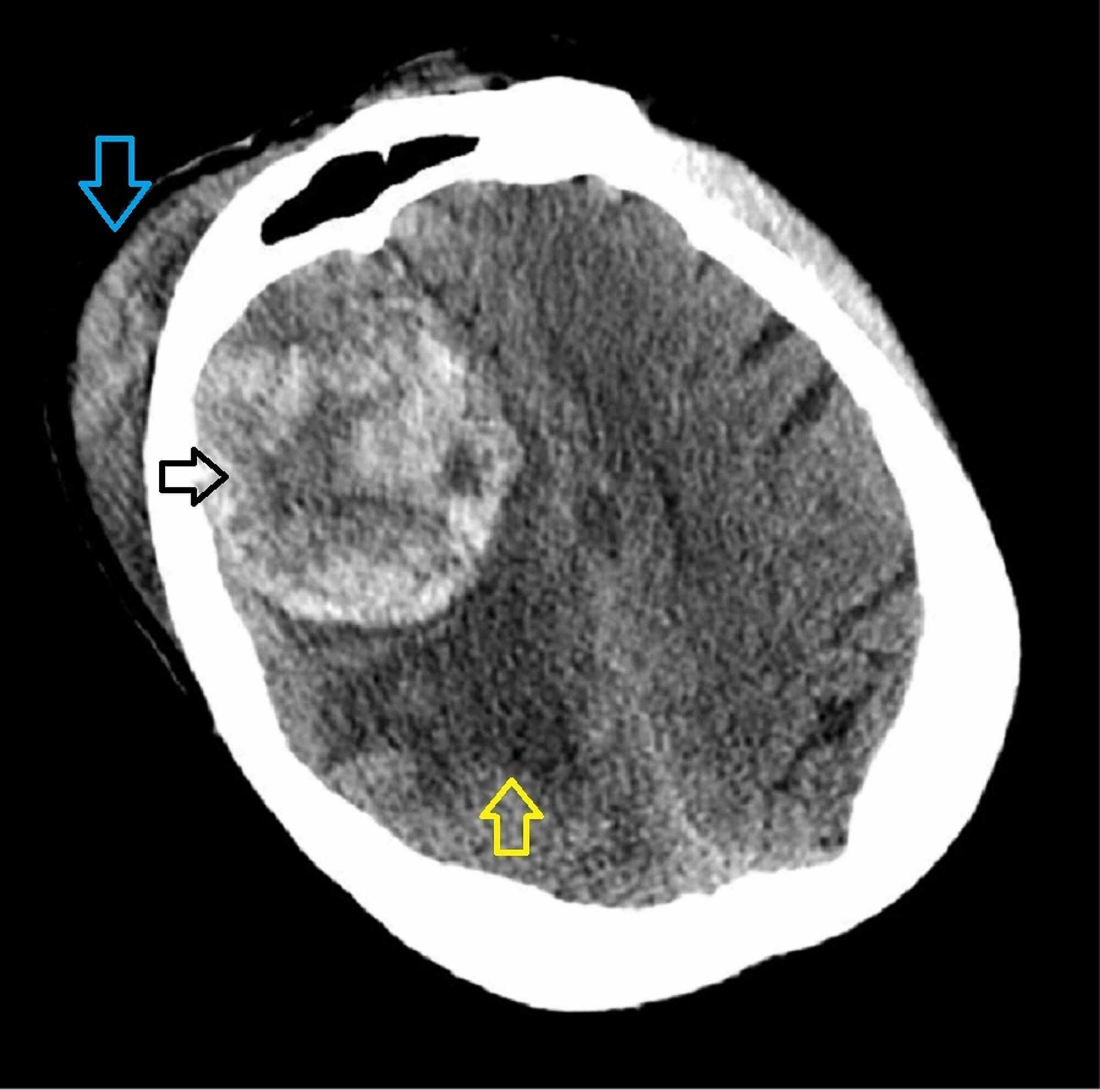
Pre-operative non-contrast CT head Axial cut at the level of the frontal sinus demonstrates a large right frontal, extra-axial, heterogeneously dense mass measuring 5.7cm x 6.5cm x 5.0cm in size (black arrow). The mass is surrounded by notable vasogenic edema (yellow arrow) causing midline shift and subfalcine herniation. A right frontal subgaleal hematoma is evident without adjacent skull fracture (blue arrow).

A 75-year-old intoxicated male with a past medical history of diabetes mellitus, hypertension, and alcohol use disorder presented to the hospital after an unwitnessed fall down one flight of stairs. Initially, the patient was measured as a Glasgow Coma Scale (GCS) score of 15 at the scene, but the patient declined neurologically to a GCS of 12 at presentation to our facility. Notably, he was drowsy, disoriented with unintelligible speech, and demonstrated left hemiplegia. Computed tomography (CT) of the head without contrast revealed a large right frontal, extra-axial, hemorrhage resulting in early uncal and subfalcine herniation (Figure 1). The hemorrhage appeared suspiciously spherical in shape with a dural base. A significant degree of adjacent vasogenic edema, out of proportion for an acute hemorrhage, was also noted thereby raising clinical concern for a possible underlying neoplasm. Adjacent bony anatomy showed no infiltrative lesion, sclerosis, or hyperostosis. Concomitant traumatic injuries included subgaleal hematoma and soft tissue edema without adjacent skull fracture.

After imaging was obtained, the patient’s level of consciousness continued to decline, requiring endotracheal intubation for airway protection. He was treated with hyperosmolar therapy en route to the operating room for emergent decompression.

Intra-operative findings

The patient was positioned using a Mayfield horseshoe device. A standard reverse question mark incision was made and a right frontal craniotomy was performed, followed by a curvilinear durotomy. A right frontal hematoma was immediately visible on the surface with gross necrotic and hemorrhagic debris evident upon entry. During evacuation of the interior of the hematoma, a thick fibrous surrounding capsule was encountered, and specimens were sent for histopathologic analysis. Additionally, a traumatic laceration of the anterior portion of the superior sagittal sinus and an associated draining vein were encountered, which necessitated repair due to ongoing hemorrhage throughout the evacuation. Once hemostasis was achieved, closure was performed in a standard multi-layer fashion without further event.

Post-operative course

**Figure 2:**
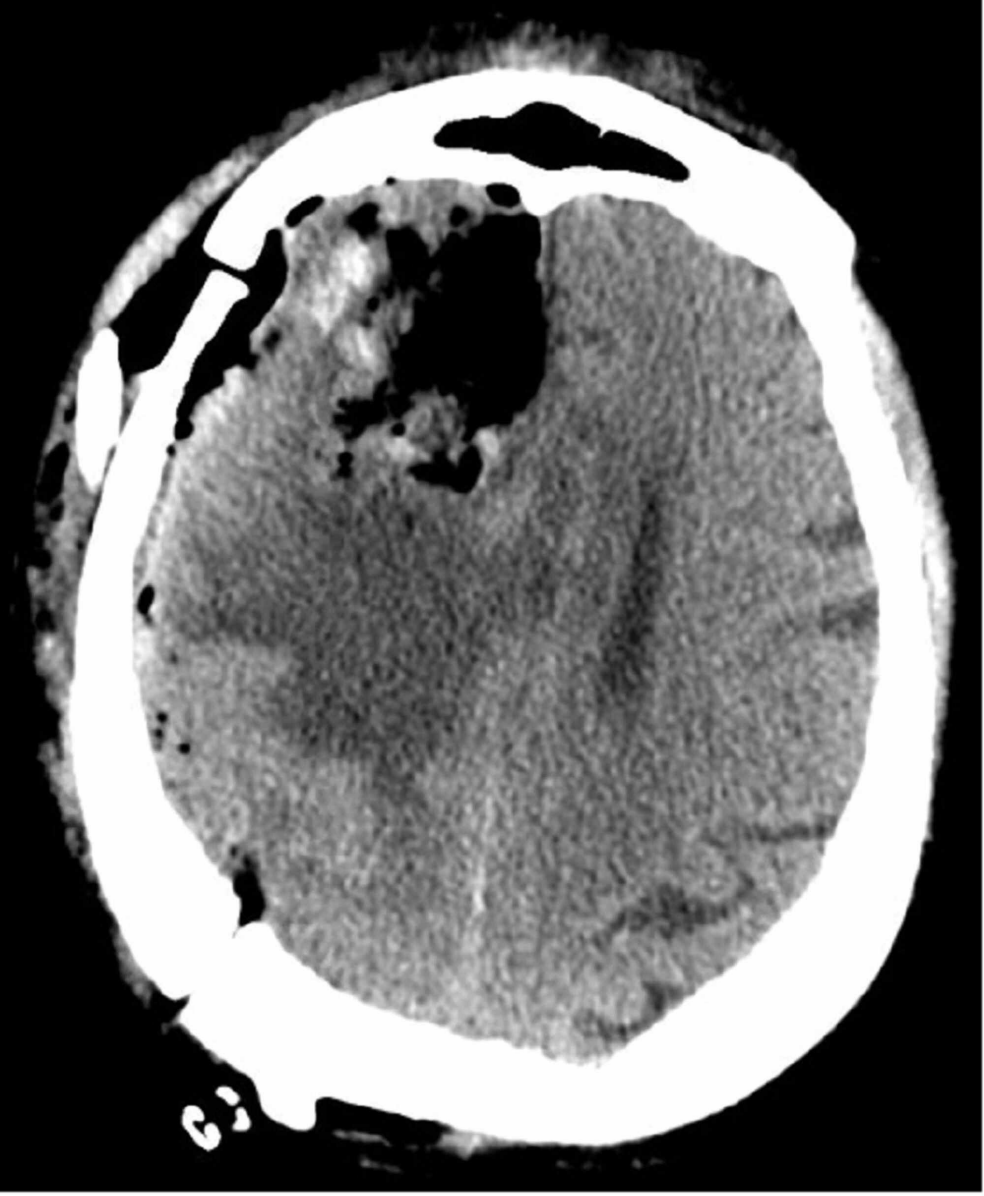
Post-operative non-contrast CT head Post-operative imaging demonstrates expected surgical change after a right frontal craniotomy with gross total resection of the right lobar hemorrhagic mass and restoration of pre-operative midline shift. Vasogenic edema appears stable.

**Figure 3:**
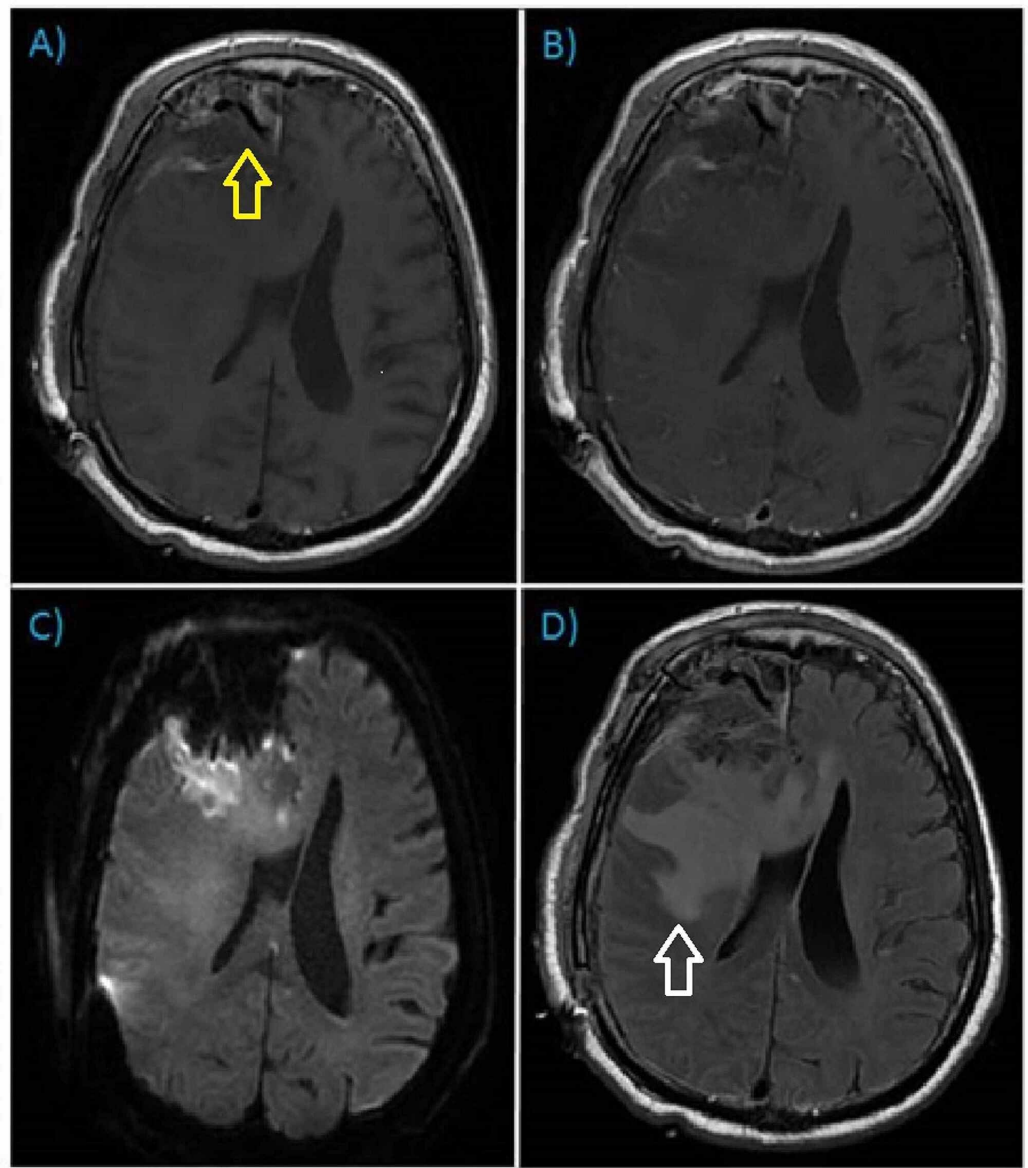
Post-operative MRI brain with and without gadolinium Post-operative MRI brain with and without contrast, axial orientation. A) T1-weighted pre-contrast; B) T1-weighted post-contrast; C) Diffusion Weighted Imaging (DWI); D) T2-weighted Fluid Attenuation Inversion Recovery (FLAIR). Post-surgical changes evident (yellow arrow) with re-demonstration of vasogenic edema (white arrow) and no residual enhancing mass.

**Figure 4:**
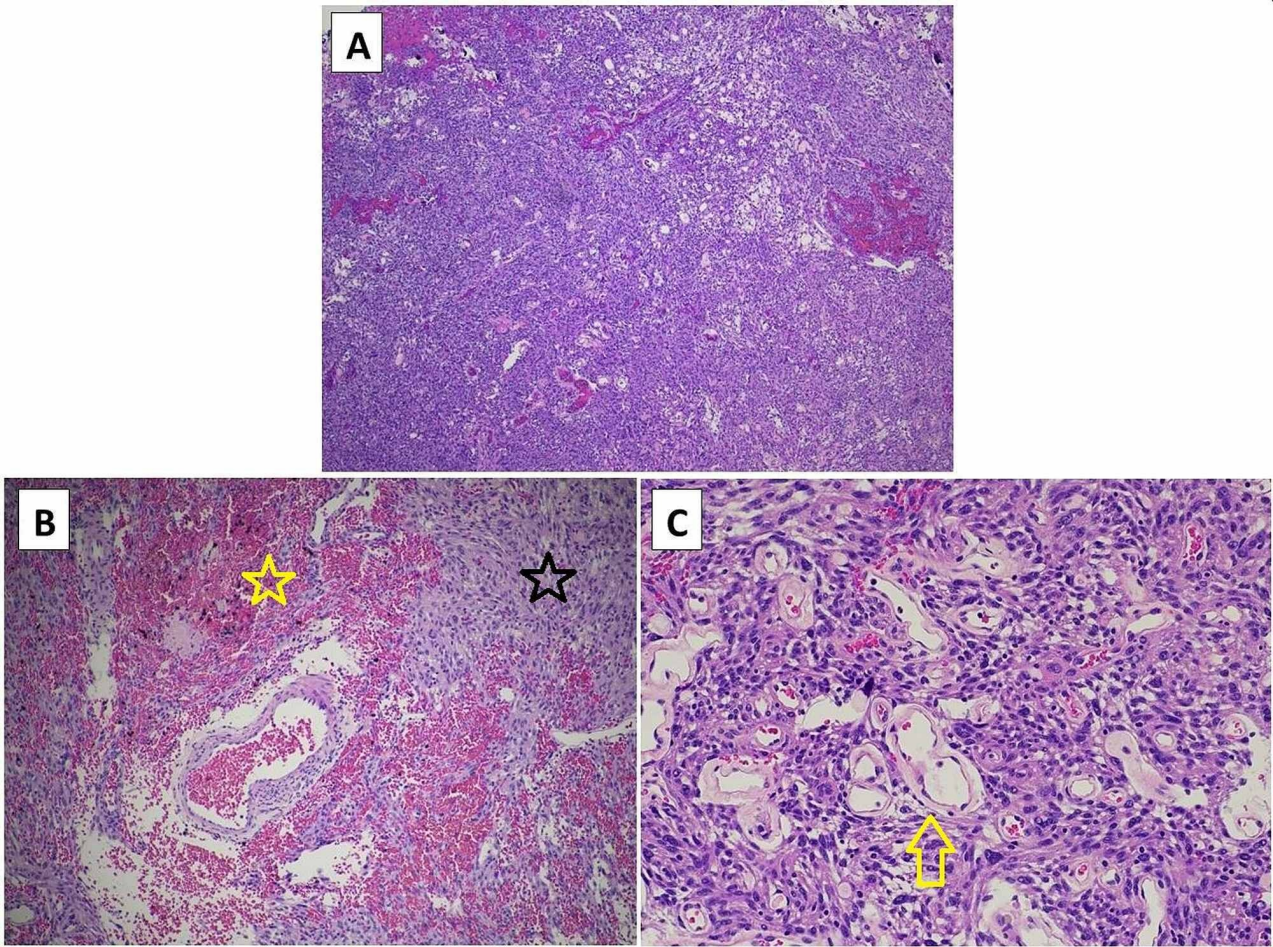
Histopathology A) Low-powered magnification demonstrating high cellularity of meningoendothelial origin, microcystic architecture, and >50% vasculature, B) Intermediate-powered image further magnifying extent of vascularity and hemorrhage (yellow star) in a spindle-celled background (black star). C) High-powered magnification demonstrating dominant hyalinized vessels (yellow arrow), microcysts, nuclear pseudoinclusions, and absence of mitotic activity.

The patient was taken to the intensive care unit in stable condition. Post-operative CT head without contrast demonstrated successful resection of the lesion and related hemorrhage (Figure 2). Post-operative magnetic resonance imaging (MRI) did not reveal residual neoplasm; however, persistent vasogenic edema remained. A gross total resection was felt to be achieved (Figure 3). Pathology confirmed diagnosis of World Health Organization (WHO) Grade 1 meningioma with focal microcystic and angiomatous pattern (Figure 4). There was no evidence of brain invasion. Tumor sections were positive for epithelial membrane antigen (EMA) and 2% Ki67 staining. In the interim, neurological exam improved to full strength on the patient’s left side. The only focal deficit evident post-operatively was a mild left facial droop. The remainder of the patient’s hospital stay was uneventful, and he was discharged to an acute rehabilitation facility on post-operative day four.

## Discussion

The patient described in this case report exhibits several proposed risk factors for hemorrhage including trauma, hypertension, diabetes mellitus, hematologic dyscrasia secondary to alcohol use disorder, convexity tumor location, and angiomatous histopathology. Given the evidence of a frontal subgaleal hematoma signifying the exact location of impact, and the directly underlying hematoma, we speculate that the opposing acceleration/deceleration forces and the tethering of the dense meningioma mass sheared bridging and intratumoral vessels, causing the hemorrhage. The intraoperative presence of the sagittal sinus and bridging vein laceration support this this hypothesis. Given the microcystic angiomatous pattern of the meningioma, and other pre-existing risk factors, the bleeding was perpetuated.

While the meningioma itself was moderate in size, and the degree of trauma was otherwise considered mild, the combination of hemorrhage in the setting of significant pre-existing vasogenic edema caused rapid progression towards brain herniation. The phenomena described here is an excellent manifestation of the Monro-Kellie hypothesis, which suggests a rapid rise in intracranial pressure at a point where autoregulation has been exhausted. In this case, neither the tumor nor the trauma by itself would have been expected to cause such a severe clinical presentation due to regulatory mechanisms. However, the combination of tumor mass effect, pre-existing vasogenic edema, traumatic brain injury, and intratumoral hemorrhage likely overwhelmed regulatory capacity, thus causing rapid neurological decline.

It is important to note that only one case report attributes the etiology of their hemorrhagic meningioma to minor head trauma whereas the majority of studies emphasize nontraumatic risk factors. Thus, while listed as a potential risk factor in several studies, trauma has since been considered a “trivial” etiology [5]. The pattern of injury in our patient, including both the sinus laceration and the overlying subgaleal hematoma in perfect opposition to the intracranial hemorrhage, appears to definitively prove a traumatic etiology. Fortunately, our patient underwent decompression with clot and tumor evacuation in a timely manner and has since recovered to previous neurological baseline.

Within the literature, most of the hemorrhagic meningiomas presented with either an extratumoral component only or combined extra-and intratumoral hemorrhage [8,9]. Examples of extratumoral hemorrhage included isolated subdural hematoma (SDH) [10-12], or subarachnoid hemorrhage (SAH) [13], whereas many case reports detail a small foci of SDH or SAH in addition to partial intratumoral hemorrhage [12,14,15]. When presenting in this manner, the pattern of hemorrhage tended to preserve some visualization of the underlying neoplasm as well as precipitate a less emergent neurological decline thereby allowing for a more definitive oncologic work-up [8,15]. Only two additional case studies demonstrate a nearly complete intratumoral hemorrhage similar to this case report, in which the tumors seemingly transform into a complete intracerebral hemorrhage thus limiting preoperative identification of neoplasm [16]. In this case report, the mechanism of the hematoma progressed to include the entire tumor volume, which is seemingly unique. We speculate that the injury to the primary venous outflow and the parasagittal bridging vein increased the intratumoral vascular congestion in the setting of the microcystic and angiomatous histopathology. This combination of acute vascular congestion and hypervascular histopathology were conducive to diffuse, rather than focal, intratumoral hemorrhage. 

The radiographic pattern of hemorrhage on initial presentation is important to scrutinize to maintain an accurate differential diagnosis and avoid delayed treatment. Early diagnosis and appropriate treatment strategy can decrease the mortality rate as low as 13.9% for hemorrhagic meningiomas [8]. When significant intratumoral hemorrhage obscures a possible underlying neoplasm, clinicians should evaluate noncontrasted CT for the following features: bony alterations including hyperostosis or erosive changes, focal cerebral and venous sinus distortion, perifocal edema, variability in signal density within the hemorrhagic area, round shape, and a clear margin between hemorrhage and adjacent parenchyma to suggest intra-lesional hemorrhage versus spontaneous intracranial hemorrhage [11]. The appearance of vasogenic edema, spherical hemorrhage, and heterogeneous density raised suspicion for neoplasm in this case, thus surgical decompression implemented a complete clot evacuation and inspection for abnormal tissue versus craniectomy alone. While MRI was the next step in diagnosis, impending herniation precluded further time-intensive testing. Most cases within the literature demonstrated a less emergent work-up and need for treatment, thus MRI was employed to further characterize the underlying meningioma and aid in surgical resection planning. Post-operatively, it will be crucial to monitor our patient with serial MRI to evaluate for both tumor recurrence as well as repeat hemorrhage. Though recurrent hemorrhage is also considered rare, there are no consistent clinical or histopathologic features that help predict its occurrence rendering close observation the neurosurgeon’s best tool [15]. Finally, performing a complete hematologic and systemic examination in addition to alcohol counseling is necessary given his extensive risk factors for hemorrhage.

The pathology ultimately resulted as a WHO Grade I meningioma. Interestingly, the histology of this case featured angiomatous morphology, a variant which makes up 2% of all meningiomas and is defined as having meningothelial cells wrapped around blood vessels in greater than 50% of the total tumor area [17]. An increased frequency of friable vessels surrounded by neoplastic cells portends an increased risk of hemorrhage, and Helle and Conley calculated the “relative bleeding tendency” of meningiomas which was increased in those tumors of angioblastic histology [4]. One should note that the term “angioblastic” is no longer recognized as a WHO subtype of meningioma and in fact refers instead to the modern-day classification of hemangiopericytoma [18]. The overall prevalence of hemorrhagic meningioma is likely overestimated in the setting of multiple previous studies including this variant and subsequent updates of the WHO classification system that render the subtype as a discrete neoplastic entity. This nuance further validates the rarity of this presentation. Finally, while the angiomatous, transitional, and meningothelial subtypes have most frequently been reported in cases with hemorrhage [4,8], no statistically significant association to histologic subtype has been proven in the literature [2,19]. A study by Wang et al. demonstrated that a greater proportion of CD31+/CD34- undifferentiated blood vessels exist in meningiomas that present with hemorrhage, however, no correlation was found for the number of differentiated vessels or total number of vessels between hemorrhagic and non-hemorrhagic tumors [19]. Despite the lack of statistical significance with regard to histologic subtype, the case presented herein adds an additional report of angiomatous meningioma presenting with intratumoral hemorrhage.

## Conclusions

Meningiomas may clinically manifest by acute intratumoral hemorrhage in the setting of moderate head trauma, particularly when the traumatic force is adjacent to the tumor location. Associations with alcoholism, proximity to dural venous sinuses, and angiomatous pathology may increase risk and extent of hemorrhage. In cases where emergent decompression precludes obtaining pre-operative MR analysis, careful scrutiny of CT imaging for characteristics of underlying neoplasm and generous hematoma evacuation are warranted. Given the increased rate of mortality in the literature, earlier diagnosis and definitive surgery is crucial.
